# Entwicklungsansätze für Impfstoffe gegen Hepatitis-C-Virus-Infektionen

**DOI:** 10.1007/s00103-021-03477-9

**Published:** 2022-01-11

**Authors:** Dorothea Bankwitz, Thomas Krey, Thomas Pietschmann

**Affiliations:** 1 Twincore Zentrum für Experimentelle und Klinische Infektionsforschung, Institut für Experimentelle Virologie, Feodor-Lynen-Str. 7, 30625 Hannover, Deutschland; 2 Medizinische Hochschule Hannover, RESIST Exzellenzcluster EXC2155, Hannover, Deutschland; 3 Zentrum für Strukturbiologie und Zellbiologie in der Medizin, Institut für Biochemie, Universität Lübeck, Lübeck, Deutschland; 4 Deutsches Zentrum für Infektionsforschung (DZIF), Partnerstandort Hamburg-Lübeck-Borstel-Riems, Braunschweig, Deutschland; 5 Institut für Virologie, Medizinische Hochschule Hannover, Hannover, Deutschland; 6 Deutsches Zentrum für Infektionsforschung (DZIF), Partnerstandort Hannover-Braunschweig, Braunschweig, Deutschland

**Keywords:** Hepatitis-C-Virus, Antikörperantwort, T‑Zellantwort, Impfstoffplattformen, Impfstoffvalidierung, Hepatitis C virus, Antibody response, T‑cell response, Vaccine platforms, Vaccine validation

## Abstract

Mehr als 10 Jahre nach der Zulassung der ersten direkt wirkenden antiviralen Wirkstoffe zur Behandlung der Hepatitis C bleibt die Inzidenz der Hepatitis-C-Virus-(HCV-)Infektion ungebrochen hoch. In manchen Ländern stecken sich mehr Menschen neu mit dem Virus an, als Patienten durch eine erfolgreiche Therapie geheilt werden. Die Entwicklung eines prophylaktischen Impfstoffes könnte die Transmission des Virus unterbinden und dadurch einen wesentlichen Beitrag zur Kontrolle dieser weltweit verbreiteten Infektion leisten. In diesem Artikel werden die besonderen Herausforderungen und die aktuellen Ansätze der HCV-Impfstoffentwicklung dargestellt.

HCV ist ein hochgradig diverses und wandlungsfähiges Virus, das zumeist dem Immunsystem entkommt und chronische Infektionen etabliert. Andererseits heilt die HCV-Infektion bei bis zu einem Drittel der exponierten Individuen aus, sodass eine schützende Immunität erreichbar ist. Zahlreiche Untersuchungen zu den Determinanten einer schützenden Immunität gegen HCV zeichnen ein immer kompletteres Bild davon, welche Ziele ein Impfstoff erreichen muss. Sehr wahrscheinlich werden sowohl starke neutralisierende Antikörper als auch wirkungsvolle zytotoxische T‑Zellen gebraucht, um sicher vor einer chronischen Infektion zu schützen. Die Schlüsselfrage ist, welche Ansätze besonders breit wirksame Antikörper und T‑Zellen heranreifen lassen. Dies wird erforderlich sein, um vor der großen Fülle unterschiedlicher HCV-Varianten zu schützen. Die jüngsten Erfolge von mRNA-Impfstoffen öffnen neue Türen auch für die HCV-Impfstoffforschung. Kombiniert mit einem tieferen Verständnis der Struktur und Funktion der viralen Hüllproteine, der Identifizierung kreuzprotektiver Antikörper- und T‑Zellepitope sowie der Nutzung standardisierter Verfahren zur Quantifizierung der Wirksamkeit von Impfkandidaten ergeben sich neue Perspektiven für die Entwicklung eines Impfstoffes.

## Abstract (en)

The incidence of hepatitis C virus (HCV) infections remains high even more than 10 years after approval of the first direct-acting antivirals for treatment of hepatitis C. In some countries, more people are newly infected with the virus than patients cured by antiviral therapy. The development of a prophylactic vaccine could prevent virus transmission and thereby make a significant contribution to control the global burden of this disease. In this article, we review the unique challenges and current approaches to HCV vaccine development.

HCV is a highly diverse and versatile virus that mostly escapes the immune system and establishes chronic infections. However, up to one third of the exposed individuals can spontaneously resolve HCV infections, which indicates that protective immunity can be achieved. Numerous studies on determinants of protective immunity against HCV show an increasingly complete picture of what a vaccine must achieve. It is very likely that both strong neutralizing antibodies and powerful cytotoxic T cells are needed to reliably protect against chronic HCV infection. The key question is which approaches allow maturation of particularly broadly effective antibodies and T cells. This will be necessary to protect against the high number of different HCV variants. The recent successes of mRNA vaccines open new doors for HCV vaccine research and development. Combined with a deeper understanding of the structure and function of the viral envelope proteins, the identification of cross-protective antibody and T‑cell epitopes as well as the use of standardized methods to quantify the effectiveness of vaccine candidates, new perspectives arise for the development of a vaccine.

## Einleitung

Hepatitis-C-Virus(HCV)-Infektionen lassen sich durch antivirale Medikamente sehr effizient behandeln. Trotzdem ist davon auszugehen, dass erst die Entwicklung eines HCV-Impfstoffs die von der Weltgesundheitsorganisation (WHO) angestrebte Elimination des Virus sicherstellen kann. Die Notwendigkeit eines präventiven HCV-Impfstoffs wird bei der Betrachtung epidemiologischer Kennzahlen deutlich. Weltweit leben 71 Mio. Menschen mit einer HCV-Infektion [1], hiervon leben 5,9 Mio. in der Europäischen Union, wobei es hier zwischen den einzelnen Ländern große Unterschiede in den Prävalenzen gibt [2]. So liegt die HCV-Seroprävalenz in der Allgemeinbevölkerung in Italien bei 5,9 %, in Deutschland hingegen nur bei 0,4 % [2].

Etwa 70 % der HCV-Infektionen heilen nicht spontan aus, sondern persistieren. Unterbleiben die Diagnose und eine Therapie, können schwere Spätfolgen auftreten und viele dieser Patienten entwickeln nach Jahrzehnten eine Leberzirrhose oder Leberkrebs. In Industrieländern werden Schätzungen zufolge 60 % der Leberzellkarzinome durch eine chronische Hepatitis C verursacht. In Europa wird bei 63 % der Lebertransplantationen als Indikation eine HCV-Infektion angegeben [3]. Im Jahr 2019 starben weltweit etwa 290.000 Menschen an Hepatitis-C-bedingten Spätfolgen [1].

Für das Erreichen der von der WHO angestrebten HCV-Elimination gibt es die Zielvorgabe, die HCV-Inzidenz bis 2030 um 80 % gegenüber der Inzidenz des Jahres 2015 zu senken (1,75 Mio. HCV-Neuinfektionen im Jahr 2015 [4]). Dieses Ziel ist ohne einen wirksamen HCV-Impfstoff schwer erreichbar [5]. Ein wichtiger Grund hierfür ist die ungebrochen hohe Rate an HCV-Neuinfektionen. Gründe hierfür sind beispielsweise die steigende Anzahl injizierender Drogenkonsumenten bedingt durch die anhaltende Opioidkrise in Nordamerika sowie durch nosokomiale Infektionen in Entwicklungs- und Schwellenländern. Im Jahr 2017 wurden 1,5 Mio. HCV-Patienten mit direkt wirkenden antiviralen Substanzen behandelt, im gleichen Jahr gab es jedoch 1,6 Mio. Neuinfektionen [6].

Im Jahr 2019 haben sich weltweit 1,5 Mio. Menschen neu mit HCV infiziert, es ist somit kein wesentlicher Rückgang bei den Neuinfektionen zu verzeichnen [1]. Nach Schätzungen der WHO waren im Jahr 2019 nur 21 % aller HCV-positiven Patienten diagnostiziert und in dieser Gruppe haben 63 % eine Behandlung mit antiviralen Substanzen erhalten [1]. Während der COVID-19-Krise war der Zugang zur HCV-Diagnostik und -Behandlung für viele Patienten weltweit stark eingeschränkt. Aufgrund dessen ist davon auszugehen, dass es zu einem Anstieg der HCV-Prävalenz kommen wird [1, 7].

Die HCV-Infektionen sind in der Bevölkerung ungleich verteilt. HCV ist hyperendemisch in der Gruppe der injizierenden Drogenkonsumenten; hier liegt die HCV-Seroprävalenz in Deutschland bei 68 % und in 25 weiteren Ländern zwischen 60 % und 80 % [8, 9]. Bei Männern, die Sex mit Männern haben, liegt die Prävalenz einer HCV-Infektion bei 3,4 % [10]. Einer HCV-Mikroelimination in diesen Bevölkerungsgruppen steht erschwerend gegenüber, dass sich erfolgreich therapierte Patienten erneut mit HCV infizieren können [11]. Wenn Personen, die intravenösen Drogenkonsum praktizieren, eine erfolgreiche HCV-Therapie durchlaufen haben und weiterhin Drogen injizieren, beträgt die HCV-Reinfektionsrate nach 6 Monaten 12,6 % und nach 18 Monaten 17,1 % [12]. Für die Sicherstellung eines Therapieerfolgs wäre der Einsatz eines präventiven Impfstoffs direkt im Anschluss an die Behandlung erforderlich. Für seronegative Personen mit intravenösem Drogenkonsum zeigen Modellierungsstudien einen signifikanten Impfeffekt, wenn bei hoher Impfquote ein Impfstoff mit 30 % Wirksamkeit verabreicht würde [13, 14].

Direkt wirkende antivirale HCV-Medikamente sind hocheffektiv, jedoch kann trotz Heilung einer HCV-Infektion die Lebererkrankung fortschreiten und Leberkrebs entstehen [15]. In der akuten Infektionsphase verläuft die HCV-Infektion in aller Regel mild und bleibt häufig unbemerkt. Deswegen wird eine Infektion vielfach erst erkannt, wenn bereits ein erheblicher Organschaden entstanden ist. Somit wäre die Prävention einer chronischen HCV-Infektion deren Behandlung deutlich vorzuziehen, da die Spätfolgen der chronischen Infektion vermieden würden. Ein HCV-Impfstoff, der zwar die akute Ansteckung nicht verhindert, aber wirksam chronische Infektionsverläufe abwendet, wäre bereits von hohem medizinischen und gesellschaftlichen Wert. Da schwere Leberschäden fast immer erst nach langjähriger HCV-Infektion auftreten, würde ein solcher Impfstoff die HCV-Morbidität und -Mortalität wesentlich senken.

Aufgrund seiner außerordentlichen Diversität und Wandlungsfähigkeit sowie verschiedener viraler Immunevasionsmechanismen ist HCV ein schwieriges Ziel für die Entwicklung eines Impfstoffes. Darüber hinaus bestehen Limitationen bei der Prüfung von Impfstoffkandidaten: Derzeit sind keine robusten Tiermodelle verfügbar, um den Impfschutz vor einer HCV-Ansteckung direkt zu überprüfen. Präzise Immunkorrelate für eine breit schützende Immunität sind nicht vollends etabliert. Darüber hinaus gestalten sich klinische Wirksamkeitsstudien schwierig, da die Virusübertragung vorwiegend bei Drogenkonsumenten auftritt und der Endpunkt für den Impferfolg (die Abwendung einer chronischen Infektion) einen langen Studienzyklus erfordert.

In diesem Übersichtsartikel werden die besonderen Herausforderungen bei der HCV-Impfstoffentwicklung aufgezeigt und die aktuellen Ansätze für die Entwicklung eines Impfstoffs dargestellt.

## Diversität, Wandlungsfähigkeit und Evasionsmechanismen von HCV

### Hohe genetische Diversität

Die 7 bekannten HCV-Genotypen (Gt) und die mehr als 67 Subtypen haben eine ungewöhnlich hohe genetische Diversität, welche sogar die genetische Vielfalt des humanen Immundefizienzvirus 1 (HIV-1) übersteigt. HCV-Genotypen unterscheiden sich in ca. 30 % der Aminosäuren, bei Subtypen sind es ca. 15 % [16]. Diese große Bandbreite von Virusvarianten macht es äußerst schwer, einen Impfstoff zu entwickeln, der vor jedem dieser HCV-Typen schützt. Die hohe Diversität von HCV wird durch 2 grundlegende Mechanismen erzeugt: Einerseits erreicht HCV eine sehr hohe Replikationsrate und kann an einem einzigen Tag in einem infizierten Patienten zwischen 10 Mrd. und 1000 Mrd. neue Viren bilden [17]. Die Genome dieser Viren werden von einer RNA-Polymerase produziert, die eine verhältnismäßig hohe Fehlerrate hat. Dies sorgt dafür, dass etwa jedes 25. neue HCV-Genom eine Mutation trägt [18]. Einige dieser Varianten entkommen den T‑Zell- und Antikörperantworten infizierter Patienten, sodass sich eine chronische Infektion etablieren und persistieren kann. Weltweit entsteht auf diese Weise ein sehr breites Spektrum von Viren, denen schwer mit einem einzigen Impfstoff Herr zu werden ist.

### Struktur und Funktion der Hüllproteine

Neben der genetischen Diversität stellen auch die besondere Struktur und Funktion der HCV-Hüllproteine die Impfstoffentwicklung vor besondere Herausforderungen [19]. Die Hüllproteine stellen den Kontakt zu Rezeptoren auf der Oberfläche von Leberzellen her und vermitteln die Aufnahme in die Zellen. Sie sind das Ziel für neutralisierende Antikörper und somit von essenzieller Bedeutung für die Entwicklung eines Impfstoffes, der vor allem über Antiköper seine Wirksamkeit entfaltet. Inzwischen wurden Teile der Struktur des viralen Hüllproteins E2 [20, 21] aufgeklärt und auch einige breit neutralisierende Antikörper aus HCV-Patienten isoliert. Viele der hochwirksamen Antikörper binden an einen Bereich des Proteins E2, der als „front layer“ bezeichnet ist. Dieser Teil des Proteins ist sehr gut zwischen unterschiedlichen Virustypen konserviert und wird für den Kontakt des Virus mit dem Rezeptor CD81 benötigt. In einer kürzlich veröffentlichten Studie wurden die Virusevolution und die induzierten Antikörper kurz vor der Ausheilung im Detail analysiert. Dabei stellte sich heraus, dass gleichzeitig mit der Bildung von wirksamen Antikörpern gegen die Front-Layer-Hüllproteinregion in genau dieser Region Mutationen akkumulierten, welche die Fähigkeit des Virus, CD81 für den Zelleintritt zu nutzen, deutlich verringert haben. Diese Beobachtung legt nahe, dass der antikörpervermittelte Immundruck Virusmutationen hervorgerufen hat, die wesentliche Einbußen in der Virusfitness verursacht haben und auf diese Weise zur spontanen Ausheilung beigetragen haben. Darüber hinaus wurde auch in einem Mausmodell gezeigt, dass Antikörper gegen die virale CD81-Bindestelle zur Ausheilung einer HCV-Infektion führen können [22]. Aus diesen Gründen bietet sich diese Region des HCV-E2-Proteins als Ziel für die Impfstoffentwicklung an.

Allerdings werden solche Antikörper auf natürlichem Wege offenbar nicht häufig genug gebildet oder sie binden nicht ausreichend gut, um das Virus aufzuhalten. Die Gründe hierfür sind im Detail nicht bekannt. Strukturbiologische Untersuchungen legen nahe, dass die CD81-Bindestelle des Virus, und damit die Hauptzielregion für hochwirksame Antikörper, strukturell sehr flexibel ist und deswegen unterschiedliche Konformationen einnehmen kann [19]. Dies könnte die Immunogenität dieser Proteinbereiche wesentlich einschränken. Andererseits scheint der N‑terminale Teil des Hüllproteins E2 ausgesprochen immunogen zu sein und die Bildung vieler Antikörper auszulösen [23]. Allerdings ist dieser Teil des Proteins funktionell offenbar gegenüber Mutationen äußerst tolerant. Als Ausdruck dieser beiden Phänomene ist das Virusgenom an dieser Stelle, der hypervariablen Region 1 (HVR1), so variabel wie an keiner anderen Position. Demzufolge nutzt das Virus die HVR1 förmlich als „Köder“ für das Immunsystem und lenkt damit von den hoch konservierten und empfindlichen Teilen, die für die Bindung von CD81 entscheidend sind, ab.

Infektionsversuche zeigen, dass die HVR1 sogar ganz deletiert werden kann, ohne dass das Virus seine Infektiosität einbüßt [24, 25]. Diese veränderten Viren reagieren wesentlich empfindlicher auf Antikörper gegen die CD81-Bindestelle als HCV-Wildviren. Deswegen geht man davon aus, dass die HVR1 den Zugang zu den hoch konservierten Bereichen förmlich abdeckt. Eine wichtige Herausforderung für die Impfstoffentwicklung ist es also, die Struktur und Funktion der HCV-Hüllproteine noch besser zu verstehen, um Antigene konstruieren zu können, welche die Ablenkung des Immunsystems auf wenig schützende Bereiche überwinden und die Bildung großer Mengen von breit neutralisierenden Antikörpern ermöglichen.

### Variable Präsentation von viralen Peptiden

Die HCV-Nichtstrukturproteine NS3, NS4A, NS4B, NS5A und NS5B, welche neue Virusgenome herstellen, sind nicht Bestandteil der Viruspartikel, sodass Antikörper gegen diese viralen Proteine für die Immunkontrolle eine untergeordnete Rolle spielen. Allerdings werden Peptide dieser Proteine auf der Oberfläche von Zellen präsentiert und durch T‑Zellen erkannt. Funktioniert diese Erkennung, wird die Zelle als infiziert gewertet und abgetötet. Impfstoffe mit diesen Viruskomponenten machen sich dieses Prinzip der zellvermittelten Immunität zunutze. Allerdings limitiert auch hier die große Diversität von HCV die Bandbreite des Immunschutzes, da die für die Erkennung entscheidenden Peptide von HCV-Genotyp zu HCV-Genotyp unterschiedlich sind [26]. Darüber hinaus unterscheiden sich Menschen hinsichtlich ihrer HLA-Allele (Genvarianten, die Merkmale des humanen Leukozytenantigensystems bestimmen), die für die Präsentation viraler Peptide verantwortlich sind, sodass nicht jeder Mensch dieselben Peptide eines Virus erfolgreich präsentiert.

Vermutlich aufgrund dieser Limitationen konnte ein Impfstoffkandidat, der diese viralen Proteine eines einzelnen HCV-Isolates codiert, in einer randomisierten, placebokontrollierten Phase-I/II-Studie die Häufigkeit chronischer HCV-Infektionen gegenüber der Kontrollgruppe nicht verringern [27]. Deswegen müssen wir mehr darüber lernen, welche T‑Zellepitope für die Erkennung besonders vieler Virustypen geeignet sind und gleichzeitig von weitverbreiteten HLA-Typen präsentiert werden können. Es ist naheliegend, dass ein Impfstoffkandidat, der sehr breit wirksame Antikörper und zellvermittelte Immunantworten induziert, besonders wirksam sein wird.

## Qualifizierung von Impfstoffen in Zellkulturen, Tiermodellen und klinischen Studien

HCV hat ein sehr enges Wirtsspektrum, nur Menschen und Schimpansen können mit HCV infiziert werden. Obwohl das Schimpansenmodell zunächst für die HCV-Forschung zur Verfügung stand, wird es wegen ethischer Bedenken seit vielen Jahren nicht mehr eingesetzt. Somit steht die HCV-Impfstoffentwicklung vor der großen Herausforderung, kein aussagekräftiges, immunkompetentes Tiermodell für die präklinische Testung von Impfstoffkandidaten zu haben. Mäuse sind in der biomedizinischen Forschung von sehr hohem Nutzen, lassen sich jedoch nicht mit HCV infizieren. Leberchimäre Mäuse, die durch die Transplantation von humanem Lebergewebe für HCV empfänglich werden, haben sich für viele Fragestellungen in der HCV-Forschung als wertvolles Modell erwiesen [28]. Für die Testung eines präventiven Impfstoffs sind sie jedoch nur bedingt nützlich, da sie immundefizient sind, um die Abstoßung des humanen Lebergewebes zu verhindern. Deswegen eignen sich diese komplizierten Tiere zwar für die Prüfung der Wirksamkeit von Antikörpern in Rahmen passiver Immunisierungsversuche; die Testung aktiver Impfstoffe, inklusive der Klärung der Frage, ob solche Impfansätze vor einer Virusansteckung schützen, ist in diesen Tieren jedoch nicht durchführbar. Vor diesem Hintergrund gibt es seit Langem Bemühungen, immunkompetente und für HCV-empfängliche Mausmodelle zu entwickeln [29]. Die Erfolgsaussichten sind schwierig abzuschätzen und es bleibt abzuwarten, ob sich hierdurch neue Möglichkeiten für die *In-vivo*-Testung von HCV-Impfstoffkandidaten ergeben.

Parallel wurden in den vergangenen Jahren vermehrt Anstrengungen unternommen, HCV-Surrogatmodelle zu entwickeln, welche auf tierpathogenen, HCV-verwandten *Hepaciviren* beruhen [30]. Das *Rodent Hepacivirus* (RHV) wurde aus wild lebenden Ratten isoliert und löst in Laborversuchen akute und chronisch persistierende Infektionen bei Laborratten aus [31]. Labormäuse können ebenfalls infiziert werden, kontrollieren die Infektion allerdings in der akuten Infektionsphase. Chronische Verläufe treten nur dann auf, wenn das Immunsystem der Mäuse unterdrückt wird. Diese interessanten Ergebnisse zeigen neue Optionen auf, die Immunabwehrmechanismen, welche vor hepaciviralen Infektionen schützen, aufzuklären. Wie hilfreich diese Modelle für die Qualifizierung von HCV-Impfstoffkandidaten sein werden, bleibt abzuwarten. Obwohl es viele Ähnlichkeiten zwischen RHV und HCV gibt, sind weitere Studien nötig, um die Vergleichbarkeit der viralen Persistenzmechanismen, Evasionsstrategien und der Mechanismen protektiver Immunität zu adressieren. Beispielsweise ist es unklar, ob RHV die entsprechenden Rattenhomologe der HCV-Eintrittsfaktoren nutzt und ob ähnliche Epitope die Virusneutralisierung durch Antikörper vermitteln. Zudem beläuft sich die Divergenz zwischen RHV und HCV im Bereich der viralen Strukturproteine auf 67–77 % auf Aminosäureebene [32].

In Ermangelung eines immunkompetenten Tiermodells sind präklinische Tests mit HCV in Zellkultur von besonderer Bedeutung, um die Wirksamkeit von Impfstoffkandidaten abzuschätzen. In Zellkulturversuchen, die zahlreiche und divergente Virustypen einschließen, können die Potenz und das Ausmaß der Kreuzneutralisierung von impfstoffinduzierten Antikörpern unter kontrollierten Bedingungen gemessen werden. Ein Vergleich mit der Wirksamkeit von Antikörpern, die während natürlicher Infektionen im Menschen auftreten, kann hergestellt werden und bietet wichtige Orientierung. Diese Arbeiten können zwar nicht klären, welche Antikörpermengen verlässlich vor einer chronischen HCV-Infektion oder gar einer Ansteckung schützen, sie bieten jedoch wichtige relative Informationen über die Qualität einzelner Kandidaten und damit deren Potenzial für eine weitere Entwicklung als HCV-Impfstoff.

Leider werden weltweit recht unterschiedliche Zellkultur-Assays verwendet, um die Wirksamkeit von Antikörpern abzuschätzen [33]. Hierbei kommen einerseits sogenannte HCV-Pseudoviren (HCVpp) und andererseits authentische, zellkulturabgeleitete Viren (HCVcc) zum Einsatz. Beide Modelle sind sehr gut geeignet, um die Wirkung von Antikörpern zu studieren. Allerdings erschwert die Nutzung verschiedener Testsysteme den Vergleich von Forschungsergebnissen unterschiedlicher Labore und damit eine rigorose Priorisierung von Impfstoffkandidaten. Demnach hat die Entwicklung standardisierter Tests für die Bewertung der Wirksamkeit von Antikörpern eine wichtige Bedeutung. Solche Testsysteme sollten die genetische und funktionelle Diversität von HCV berücksichtigen und erfordern deswegen breite vergleichende Untersuchungen mit zahlreichen Virustypen.

Solche Studien können zudem interessante Befunde über Erregereigenschaften erbringen: So hat eine vergleichende Analyse der Neutralisierung von 13 unterschiedlichen HCV-Typen durch mehr als 100 polyklonale Antikörperpräparationen von chronisch HCV-infizierten Patienten ergeben, dass sich diese Viren 6 funktionell ähnlichen Virusgruppen zuordnen lassen, welche bezüglich ihrer Interaktion mit neutralisierenden Antikörpern vergleichbare Eigenschaften haben [34]. Die Virussequenzen waren hingegen nicht geeignet, diese Einteilung, die auf ähnlichen biologischen Eigenschaften beruht, zu treffen. Weitere Untersuchungen sind erforderlich, um zu klären, ob und wie viele weitere funktionell abgrenzbare „HCV-Biotypen“ es gibt, die sich hinsichtlich ihrer Interaktion mit polyklonalen Antikörpern unterscheiden. Das Spektrum von „Referenzviren“, welches diese 6 Biotypen umfasst, bietet sich als Testsystem für die Bewertung kreuzneutralisierender Antikörper an [34].

Die standardisierte Nutzung von HCVpp- und/oder HCVcc-Neutralisierungstests gibt wesentliche Orientierung über die Wirksamkeit impfstoffinduzierter Antikörper. Diese *In-vitro*-Assays können jedoch nicht klären, ob eine Ansteckung oder eine chronische Infektion sicher verhindert würden. Vor diesem Hintergrund und auch, weil die HCV-Infektion sehr gut behandelbar ist, wird neuerdings auch die Etablierung eines kontrollierten humanen HCV-Infektionsmodells diskutiert („controlled human infection model“; CHIM; [35]). Sicher sind hier noch wichtige virologische, ethische und regulatorische Fragen zu lösen: Mit welchem Inokulum sollte infiziert werden, um eine natürliche Infektion zu simulieren? Wie kann dieses Inokulum standardisiert und sicher bereitgestellt werden? Welche Risiken gehen mit einer transienten Virusinfektion einher und gibt es Langzeitfolgen? Wie lange könnte man ethisch vertretbar den Studienzeitraum definieren, um sicher die Wirksamkeit des Impfstoffes abzuschätzen und gleichzeitig die Probanden nur möglichst kurz einer Infektion auszusetzen? Vorausgesetzt diese und weitere Fragen können im Einklang mit einer ethisch-rechtlichen Risiko-Nutzen-Abwägung geklärt werden, könnte die Verfügbarkeit eines humanen HCV-Infektionsmodells die klinische Prüfung von HCV-Impfstoffkandidaten erleichtern.

## Impfstoffkandidaten

### Virale Vektoren

Eine Reihe verschiedener Ansätze wurde bislang für die Entwicklung eines HCV-Impfstoffs herangezogen. Virale Vektoren können hochwirksame zelluläre Immunreaktionen induzieren [36] und stellen daher einen attraktiven Ansatz für die Entwicklung eines HCV-Impfstoffs dar. Dabei sind insbesondere das humane Adenovirus 6 (HuAd6), das Schimpansenadenovirus 3 (ChAd3) und das modifizierte Vacciniavirus Ankara (MVA; [37]) interessant. Allerdings ist vor dem Hintergrund der erfolgreichen Zulassung eines rVSV-Ebolavirusimpfstoffes [38] im Jahr 2019 auch das rekombinante vesikuläre Stomatitisvirus (rVSV) als viraler Vektor wieder mehr in den Fokus gerückt [39].

Die einzigen auf viralen Vektoren basierenden HCV-Impfstoffkandidaten, die in Phase I und Phase I/II an Menschen getestet wurden, waren ChAd3 und MVA, die jeweils für die HCV-Nichtstrukturproteine NS3, NS4A, NS4B, NS5A und inaktiviertes NS5B (NSmut) codieren, das aus dem Gt1b-BK-Isolat stammt [40, 41]. Diese Kandidaten wurden zusammen in einer heterologen Prime-Boost-Strategie getestet, bei der Erwachsene, bei denen aufgrund von Drogenkonsum ein Risiko für eine HCV-Infektion bestand, mit ChAd3-NSmut geprimt und 8 Wochen später mit MVA-NSmut geboostert wurden [27]. Die Ergebnisse dieser Studie zeigten, dass trotz induzierter CD4+- und CD8+-T-Zellantwort kein signifikanter Unterschied in der Inzidenz der chronischen HCV-Infektion zwischen den Gruppen beobachtet wurde.

Ein tiefgehendes Verständnis für die Gründe dieses Scheiterns wird für die weitere Strategie, einen effizienten HCV-Impfstoff zu entwickeln, entscheidend sein. Da der ChAd3/MVA-NSmut-Kandidat ausschließlich auf T‑Zellepitope abzielte, könnte das Scheitern dieses Impfstoffkandidaten darauf hindeuten, dass die humorale Immunantwort eine wichtige Rolle spielt und daher bei einem Impfstoffkandidaten in jedem Fall mitberücksichtigt werden muss.

### Inaktivierte Viruspartikel

In vielen antiviralen Impfstoffen werden virale Partikel als Impfstoffantigene verwendet [42]. Diese wirken primär über die Induktion neutralisierender Antikörper. Auch aus Zellkultur gewonnene inaktivierte HCV-Partikel (HCVcc) sind in Tiermodellen immunogen und induzieren schützende Antikörper [43–45]. Allerdings stellen die Konzentrierung und Aufreinigung von Viruspartikeln eine Herausforderung für die Impfstoffherstellung dar. In gängigen Zellkulturmodellen wächst HCV zu eher niedrigen Virustitern, was die Ausbeute limitiert und bezüglich der Skalierbarkeit der Produktion zu Limitationen führt. Zudem haben HCV-Partikel eine ähnliche Dichte, wie sekretierte humane Lipoproteine, die während der Produktion ebenfalls von den virusproduzierenden Zellen freigesetzt werden. Dies erschwert die Abtrennung von zellulären Bestandteilen und Verunreinigungen und kompliziert den Produktionsprozess.

Daher ist – wie bei vielen anderen inaktivierten Impfstoffen – die Entwicklung eines effizienten, mit industriellen Anforderungen kompatiblen Verfahrens zur Produktion des inaktivierten Impfstoffs ein wesentlicher Engpass für die Herstellung eines inaktivierten Impfstoffs aus HCVcc für den Menschen [46, 47]. Obwohl die Immunogenität inaktivierter HCV-Partikel erwiesen ist, bleibt abzuwarten, ob der hohe Produktionsaufwand in einem akzeptablen Verhältnis gegenüber anderen Kandidaten mit einfacheren Produktionsverfahren steht.

### Rekombinante Hüllproteine

Die Nutzung rekombinanter Hüllproteine ist eine vielseitige und wirtschaftliche und deswegen attraktive Alternative bei der Impfstoffherstellung. Solche *Subunit*-Vakzine werden zudem bereits erfolgreich zur Prophylaxe gegen eine Vielzahl von viralen Krankheitserregern eingesetzt [48]. Die HCV-Glykoproteine E1 und E2 sind die primären Angriffspunkte der humoralen Immunantwort und daher als Immunogen prädestiniert [49].

E1 und E2 sind Transmembranproteine vom Typ I, die ein nichtkovalentes Heterodimer bilden und auf dem reifen Viruspartikel in kovalenten Strukturen höherer Ordnung beobachtet wurden [50]. Bislang sind die meisten charakterisierten neutralisierenden Antikörper (nAks) gegen das HCV E2 gerichtet. Es gibt mehrere lineare und diskontinuierliche Regionen in HCV E2, an die nAks binden können. Die meisten dieser Epitope sind auf der als „front layer“ bezeichneten Region von E2 lokalisiert und beinhalten Teilbereiche, die für die Bindung an den Rezeptor CD81 wichtig sind.

Der bislang erfolgreichste HCV-Impfstoffkandidat war das aus einem Gt1a-Isolat (H77) gewonnene, rekombinante HCV-E1E2-Protein (rE1E2), welches nach Immunisierung von Schimpansen in 5 von 7 Fällen zu einer sterilen Immunität gegen eine homologe Infektion führte, während die verbleibenden 2 Tiere eine limitierte akute Infektion durchmachten [51]. Dasselbe Immunogen in Kombination mit dem Adjuvans MF59 stellte sich in einer Phase-I-Studie in verschiedenen Dosen als verträglich heraus [52] und induzierte in den Patienten polyfunktionale CD4+-T-Zellantworten sowie in 3 von 16 Probanden kreuzreaktive nAks [53, 54].

### Virusartige Partikel (VLPs)

Eine weitere Möglichkeit zur Präsentation von Immunogenen sind VLPs, ein vielversprechender Bereich der Impfstoffforschung, der in den Impfstoffen gegen das Hepatitis-B-Virus (HBV) und das humane Papillomavirus (HPV) bereits Marktreife erlangt hat und auch im Rahmen der HCV-Impfstoffentwicklung exploriert wurde [55]. Dabei assemblieren virale Strukturproteine genomunabhängig zu einem Partikel, welches dem Virus ähnelt, aber nicht die Fähigkeit zur Replikation besitzt. Aufgrund der Multimerisierung in einer repetitiven Struktur und ihrer Fähigkeit, in Gewebelymphknoten zu gelangen, sind VLPs in der Regel immunogener als lösliche Proteine [56, 57].

VLPs für HCV wurden zuerst in der Insektenzelllinie Sf9 mithilfe eines Baculovirus produziert, welches die Glykoproteine E1 und E2 codiert [58]. Diese VLPs induzierten eine HCV-spezifische zelluläre und humorale Immunantwort sowohl in Mäusen als auch in Pavianen [58–60]. Bei der nachfolgenden Immunisierung von Schimpansen wurde jedoch in 3 von 4 Tieren trotz einer beobachteten CD4+-und CD8+-T-Zellreaktion keine humorale Reaktion nachgewiesen [61]. Die Arbeit an diesem Impfstoffkandidaten wurde im Folgenden nicht fortgeführt.

Ein weiteres Produktionssystem für HCV-VLPs wurde kürzlich mithilfe einer Transduktion der menschlichen Hepatomzelllinie Huh‑7 mit rekombinanten Adenoviren etabliert, welche die HCV-Strukturproteine codieren [62]. Um die Diversität von HCV zu reflektieren, wurde dieses im Original für ein Gt1a-Isolat (H77) entwickelte VLP-Produktionssystem auch für Strukturproteine der Gt1b, 2a und 3a etabliert und eine Mischung der erzeugten VLPs als quadrivalenter Impfstoffkandidat charakterisiert [63–65]. Dabei zeigte sich sowohl in Mäusen als auch in Schweinen eine Induktion von nAks und die Aktivierung von CD4+- und CD8+-T-Zellreaktionen, allerdings fehlen bislang noch Daten zur Neutralisation heterologer HCV-Isolate.

Um die angesprochene verbesserte Immunogenität von multimeren Partikeln zu nutzen, wurde auch lösliches E2 auf selbstorganisierende Ferritinpartikel aufgebracht, was im Vergleich zu monomerem löslichen E2 zur Induktion erhöhter nAk-Titer führte [66]. Einer ähnlichen Strategie folgend, wurden die Glykoproteine E1, E2 oder E1E2 auch in Nanopartikel des Hepatitis-B-Virus-(HBV-)Oberflächenantigens (HBsAg) eingebaut und diese chimären HBV-HCV-Partikel induzierten in Kaninchen kreuzneutralisierende Aks gegen Gt1a, 1b, 2a und 3 [67]. In diesen Studien fiel auf, dass die humoralen Reaktionen bei Tieren, die mit E1E2-haltigen HBsAg-VLPs geimpft wurden, im Vergleich zu Tieren, die HBsAg-VLPs der einzelnen Proteine empfingen, deutlich geringer ausfielen. Dies könnte auf eine schwächere Immunogenität der Heterodimere hindeuten, z. B. durch eine Maskierung immundominanter Epitope.

## Strukturbasiertes Impfstoffdesign

In Fällen, in denen herkömmliche Impfstoffe keine oder nur eine unzureichende Immunreaktion hervorrufen, kann das Epitop konformationell eingegrenzt und dadurch stabilisiert werden, z. B. durch epitopfokussiertes Impfstoffdesign oder chemische Peptidmodifikation wie eine Zyklisierung. Eine solche Stabilisierung der Epitopkonformation kann die Antigenität verbessern. Überzeugende Ergebnisse entsprechender Studien bei ähnlichen Viren, wie dem respiratorischen Synzytialvirus (RSV; [68]), dem Influenzavirus (IAV; [69]) und dem humanen Immundefizienzvirus (HIV; [70]), haben epitopfokussiertes Impfstoffdesign auch für HCV attraktiv werden lassen.

In einer der ersten Studien solcher Art wurden Neutralisationsepitope aus E1 (Aminosäuren 314–324) oder E2 (Aminosäuren 412–423) in einer von nAks erkannten Konformation auf sogenannte Trägerproteine („scaffolds“) transplantiert und diese dann als Immunogen verwendet [71]. 3 E1- und 5 E2-Scaffolds binden die nAks IGH526 (E1) bzw. HCV1 [71], leider liegen aber bislang keine Neutralisationsdaten für die unterschiedlichen Scaffolds vor.

In einer zweiten Studie wurde eine zyklische Variante eines Epitop-I-Peptids entwickelt, die peptidspezifische, aber zumeist nichtneutralisierende Aks in Mäusen induzierte [43]. Analog dazu wurden in einer weiteren Studie 2 weitere strukturbasierte Immunogene getestet: Ein zyklisches Defensin und ein bivalentes Immunogen mit 2 Epitop-I-Kopien auf der E2-Oberfläche lösten bei Mäusen eine stärkere epitopspezifische Reaktion und eine höhere Neutralisierungseffizienz im Serum gegen homologe HCV-Infektion aus als das native Peptidepitop [72]. Allerdings war die Neutralisation heterologer HCV-Stämme nur limitiert möglich.

In einem alternativen Ansatz wurde gezeigt, dass ein antiidiotypischer Ak, also ein Antikörper, dessen Paratop ein Neutralisierungsepitop nachahmt, eine robuste nAk-Antwort induziert [73], was darauf hindeutet, dass solche strukturbasierten Strategien eine vielversprechende Alternative zur Entwicklung eines sicheren und effizienten B‑Zellimpfstoffs darstellen können.

Wie oben erwähnt, verbessert die Multimerisierung die humorale Immunantwort gegen bestimmte Antigene beträchtlich und ähnliche Strategien wurden daher auch im strukturbasierten Impfstoffdesign angewendet [68, 74]. Auch für HCV wurde eine solche Multimerisierung mit dem epitopbasierten Scaffold-Design für E1 (aa314-324) und dem E2-Epitop I kombiniert, indem 24 Kopien dieser Scaffolds auf dem multivalenten, selbstassemblierenden Ferritin aus *Helicobacter pylori* präsentiert wurden [71].

## Fazit

Hohe Diversität und ausgeprägte Wandlungsfähigkeit machen HCV zu einem schwierigen Erregerziel für die Entwicklung eines protektiven Impfstoffs. Trotz dieser Viruseigenschaften heilt die Infektion bei bis zu einem Drittel der virusexponierten Menschen aus. Dies zeigt, dass eine schützende Immunantwort gegen das Virus erreichbar ist. Ein präzises Verständnis der immunologischen Korrelate einer natürlichen Ausheilung wird wesentliche Orientierung für die Impfstoffentwicklung geben. Antikörper tragen sehr wahrscheinlich zur Ausheilung der HCV-Infektion in der akuten Infektionsphase bei [75, 76]. Welche Ziele auf den Hüllproteinen in dieser entscheidenden Frühphase der Infektion durch Antikörper abgedeckt werden müssen, ist bisher allerdings im Detail nicht bekannt. Möglich, dass es sich vorwiegend um konservierte Epitope der CD81-Bindestelle handelt. Die Klärung dieser Frage könnte eine wichtige Bedeutung für die Entwicklung optimierter Antigene haben.

Bei SARS-CoV‑2 und bei RSV konnten auf Basis struktureller Informationen optimierte Hüllproteine gestaltet werden, welche eine stabilisierte Form des Präfusionskomplexes präsentieren und große Mengen neutralisierende Antikörper induzieren. Vielleicht erlauben umfassendere strukturelle Informationen über den HCV-Hüllproteinkomplex in Zukunft eine ähnliche Optimierung der Hüllproteine, welche gezielt die Immunogenität der konservierten Hüllproteinepitope verstärkt.

Zahlreiche Studien belegen, dass robuste, multispezifische T‑Zellantworten mit einer natürlichen Ausheilung der Infektion korrelieren [76, 77]. Vermutlich können sowohl Antikörper als auch zytotoxische T‑Zellen für sich genommen eine schützende Wirkung entfalten und sehr wahrscheinlich ist der Schutz am größten, wenn gleichzeitig beide Arme der adaptiven Immunantwort wirksam sind. Allerdings variieren wichtige T‑Zellepitope zwischen den Virustypen und es ist derzeit nicht klar, welche T‑Zellepitope besonders breit schützen. Auch in diesem Bereich benötigen wir mehr Informationen über die Determinanten einer kreuzprotektiven Immunität bei HCV. Zukünftige Studien müssen klären, ob beispielsweise eine besondere Auswahl von Epitopen weniger besonderer Virustypen oder andererseits viele Sequenzvarianten eines Epitops einen breiten T‑Zellschutz aufbauen. Die jüngsten Durchbrüche bei der Entwicklung und dem Einsatz mRNA-basierter Impfstoffe geben sicherlich wesentliche und nachhaltige Impulse auch für die HCV-Impfstoffforschung. Unabhängig davon, ob diese Verabreichungsform bei HCV besondere immunologische Vorteile bietet, sollte alleine die große Flexibilität der Plattform die vergleichende Analyse neuer Impfstoffkandidaten wesentlich beschleunigen und die Forschung voranbringen.

## References

[1] WHO (2021). Global progress report on HIV, viral hepatitis and sexually transmitted infections, 2021. Accountability for the global health sector strategies 2016–2021: actions for impact.

[2] Hofstraat SHI, Falla AM, Duffell EF (2017). Current prevalence of chronic hepatitis B and C virus infection in the general population, blood donors and pregnant women in the EU/EEA: a systematic review. Epidemiol Infect.

[3] Zimmermann R, Bremer V, Kollan C, Krings A, Schmidt D, Steffen G, Dudareva S (2020). Zur Situation bei wichtigen Infektionskrankheiten in Deutschland – Hepatitis C im Jahr 2019.

[4] WHO (2017). Global hepatitis report 2017.

[5] Scott N, Wilson DP, Thompson AJ (2019). The case for a universal hepatitis C vaccine to achieve hepatitis C elimination. BMC Med.

[6] Hill AM, Nath S, Simmons B (2017). The road to elimination of hepatitis C: analysis of cures versus new infections in 91 countries. J Virus Erad.

[7] Blach S, Kondili LA, Aghemo A (2021). Impact of COVID-19 on global HCV elimination efforts. J Hepatol.

[8] Sperle I, Steffen G, Leendertz SA (2020). Prevalence of hepatitis B, C, and D in Germany: results from a scoping review. Front Public Health.

[9] Nelson PK, Mathers BM, Cowie B (2011). Global epidemiology of hepatitis B and hepatitis C in people who inject drugs: results of systematic reviews. Lancet.

[10] Jin F, Dore GJ, Matthews G (2021). Prevalence and incidence of hepatitis C virus infection in men who have sex with men: a systematic review and meta-analysis. Lancet Gastroenterol Hepatol.

[11] Martinello M, Grebely J, Petoumenos K (2017). HCV reinfection incidence among individuals treated for recent infection. J Viral Hepat.

[12] Schulkind J, Stephens B, Ahmad F (2019). High response and re-infection rates among people who inject drugs treated for hepatitis C in a community needle and syringe programme. J Viral Hepat.

[13] Scott N, Mcbryde E, Vickerman P (2015). The role of a hepatitis C virus vaccine: modelling the benefits alongside direct-acting antiviral treatments. BMC Med.

[14] Stone J, Martin NK, Hickman M (2016). The potential impact of a hepatitis C vaccine for people who inject drugs: is a vaccine needed in the age of direct-acting antivirals?. PLoS ONE.

[15] Domovitz T, Gal-Tanamy M (2021). Tracking down the epigenetic footprint of HCV-induced hepatocarcinogenesis. J Clin Med.

[16] Smith DB, Bukh J, Kuiken C (2014). Expanded classification of hepatitis C virus into 7 genotypes and 67 subtypes: updated criteria and genotype assignment web resource. Hepatology.

[17] Neumann AU, Lam NP, Dahari H (1998). Hepatitis C viral dynamics in vivo and the antiviral efficacy of interferon-alpha therapy. Science.

[18] Ribeiro RM, Li H, Wang S (2012). Quantifying the diversification of hepatitis C virus (HCV) during primary infection: estimates of the in vivo mutation rate. PLoS Pathog.

[19] Stroh LJ, Nagarathinam K, Krey T (2018). Conformational flexibility in the CD81-binding site of the hepatitis C virus glycoprotein E2. Front Immunol.

[20] Khan AG, Whidby J, Miller MT (2014). Structure of the core ectodomain of the hepatitis C virus envelope glycoprotein 2. Nature.

[21] Kong L, Giang E, Nieusma T (2013). Hepatitis C virus E2 envelope glycoprotein core structure. Science.

[22] de Jong YP, Dorner M, Mommersteeg MC (2014). Broadly neutralizing antibodies abrogate established hepatitis C virus infection. Sci Transl Med.

[23] Prentoe J, Bukh J (2018). Hypervariable region 1 in envelope protein 2 of hepatitis C virus: a linchpin in neutralizing antibody evasion and viral entry. Front Immunol.

[24] Bankwitz D, Steinmann E, Bitzegeio J (2010). Hepatitis C virus hypervariable region 1 modulates receptor interactions, conceals the CD81 binding site, and protects conserved neutralizing epitopes. J Virol.

[25] Prentoe J, Jensen TB, Meuleman P (2011). Hypervariable region 1 differentially impacts viability of hepatitis C virus strains of genotypes 1 to 6 and impairs virus neutralization. J Virol.

[26] von Delft A, Humphreys IS, Brown A (2016). The broad assessment of HCV genotypes 1 and 3 antigenic targets reveals limited cross-reactivity with implications for vaccine design. Gut.

[27] Page K, Melia MT, Veenhuis RT (2021). Randomized trial of a vaccine regimen to prevent chronic HCV infection. N Engl J Med.

[28] Burm R, Collignon L, Mesalam AA, Meuleman P (2018). Animal models to study hepatitis C virus infection. Front Immunol.

[29] Berggren KA, Suzuki S, Ploss A (2020). Animal models used in hepatitis C virus research. Int J Mol Sci.

[30] Ploss A, Kapoor A (2020). Animal models of hepatitis C virus infection. Cold Spring Harb Perspect Med.

[31] Trivedi S, Murthy S, Sharma H (2018). Viral persistence, liver disease, and host response in a hepatitis C-like virus rat model. Hepatology.

[32] Kapoor A, Simmonds P, Scheel TK (2013). Identification of rodent homologs of hepatitis C virus and pegiviruses. mBio.

[33] Bailey JR, Urbanowicz RA, Ball JK, Law M, Foung SKH (2019). Standardized method for the study of antibody neutralization of HCV pseudoparticles (HCVpp). Methods Mol Biol.

[34] Bankwitz D, Bahai A, Labuhn M (2020). Hepatitis C reference viruses highlight potent antibody responses and diverse viral functional interactions with neutralising antibodies. Gut.

[35] Liang TJ, Feld JJ, Cox AL, Rice CM (2021). Controlled human infection model—fast track to HCV vaccine?. N Engl J Med.

[36] Ura T, Okuda K, Shimada M (2014). Developments in viral vector-based vaccines. Vaccines (Basel).

[37] Barnes E, Folgori A, Capone S (2012). Novel adenovirus-based vaccines induce broad and sustained T cell responses to HCV in man. Sci Transl Med.

[38] Jones SM, Feldmann H, Stroher U (2005). Live attenuated recombinant vaccine protects nonhuman primates against Ebola and Marburg viruses. Nat Med.

[39] Case JB, Rothlauf PW, Chen RE (2020). Replication-competent vesicular stomatitis virus vaccine vector protects against SARS-coV-2-mediated pathogenesis in mice. Cell Host Microbe.

[40] Capone S, Zampaglione I, Vitelli A (2006). Modulation of the immune response induced by gene electrotransfer of a hepatitis C virus DNA vaccine in nonhuman primates. J Immunol.

[41] Swadling L, Capone S, Antrobus RD (2014). A human vaccine strategy based on chimpanzee adenoviral and MVA vectors that primes, boosts, and sustains functional HCV-specific T cell memory. Sci Transl Med.

[42] Plotkin SA, Plotkin SL (2011). The development of vaccines: how the past led to the future. Nat Rev Microbiol.

[43] Akazawa D, Moriyama M, Yokokawa H (2013). Neutralizing antibodies induced by cell culture-derived hepatitis C virus protect against infection in mice. Gastroenterology.

[44] Gottwein JM, Bukh J (2013). Viral hepatitis: cell-culture-derived HCV—a promising vaccine antigen. Nat Rev Gastroenterol Hepatol.

[45] Yokokawa H, Higashino A, Suzuki S (2018). Induction of humoural and cellular immunity by immunisation with HCV particle vaccine in a non-human primate model. Gut.

[46] Wolf MW, Reichl U (2011). Downstream processing of cell culture-derived virus particles. Expert Rev Vaccines.

[47] Nestola P, Peixoto C, Silva RR, Alves PM, Mota JP, Carrondo MJ (2015). Improved virus purification processes for vaccines and gene therapy. Biotechnol Bioeng.

[48] Plotkin SA (2005). Vaccines: past, present and future. Nat Med.

[49] Ball JK, Tarr AW, Mckeating JA (2014). The past, present and future of neutralizing antibodies for hepatitis C virus. Antiviral Res.

[50] Vieyres G, Thomas X, Descamps V, Duverlie G, Patel AH, Dubuisson J (2010). Characterization of the envelope glycoproteins associated with infectious hepatitis C virus. J Virol.

[51] Choo QL, Kuo G, Ralston R (1994). Vaccination of chimpanzees against infection by the hepatitis C virus. Proc Natl Acad Sci U S A.

[52] Frey SE, Houghton M, Coates S (2010). Safety and immunogenicity of HCV E1E2 vaccine adjuvanted with MF59 administered to healthy adults. Vaccine.

[53] Law JL, Chen C, Wong J (2013). A hepatitis C virus (HCV) vaccine comprising envelope glycoproteins gpE1/gpE2 derived from a single isolate elicits broad cross-genotype neutralizing antibodies in humans. PLoS One.

[54] Ray R, Meyer K, Banerjee A (2010). Characterization of antibodies induced by vaccination with hepatitis C virus envelope glycoproteins. J Infect Dis.

[55] Torresi J (2017). The rationale for a preventative HCV virus-like particle (VLP) vaccine. Front Microbiol.

[56] Bachmann MF, Jennings GT (2010). Vaccine delivery: a matter of size, geometry, kinetics and molecular patterns. Nat Rev Immunol.

[57] Lopez-Sagaseta J, Malito E, Rappuoli R, Bottomley MJ (2016). Self-assembling protein nanoparticles in the design of vaccines. Comput. Struct. Biotechnol. J..

[58] Baumert TF, Ito S, Wong DT, Liang TJ (1998). Hepatitis C virus structural proteins assemble into viruslike particles in insect cells. J Virol.

[59] Lechmann M, Murata K, Satoi J, Vergalla J, Baumert TF, Liang TJ (2001). Hepatitis C virus-like particles induce virus-specific humoral and cellular immune responses in mice. Hepatology.

[60] Jeong SH, Qiao M, Nascimbeni M (2004). Immunization with hepatitis C virus-like particles induces humoral and cellular immune responses in nonhuman primates. J Virol.

[61] Elmowalid GA, Qiao M, Jeong SH (2007). Immunization with hepatitis C virus-like particles results in control of hepatitis C virus infection in chimpanzees. Proc Natl Acad Sci Usa.

[62] Chua BY, Johnson D, Tan A (2012). Hepatitis C VLPs delivered to dendritic cells by a TLR2 targeting lipopeptide results in enhanced antibody and cell-mediated responses. PLoS One.

[63] Christiansen D, Earnest-Silveira L, Chua B (2018). Immunological responses following administration of a genotype 1a/1b/2/3a quadrivalent HCV VLP vaccine. Sci Rep.

[64] Earnest-Silveira L, Chua B, Chin R (2016). Characterization of a hepatitis C virus-like particle vaccine produced in a human hepatocyte-derived cell line. J Gen Virol.

[65] Christiansen D, Earnest-Silveira L, Grubor-Bauk B (2019). Pre-clinical evaluation of a quadrivalent HCV VLP vaccine in pigs following microneedle delivery. Sci Rep.

[66] Yan Y, Wang X, Lou P (2020). A nanoparticle-based hepatitis C virus vaccine with enhanced potency. J Infect Dis.

[67] Beaumont E, Patient R, Hourioux C, Dimier-Poisson I, Roingeard P (2013). Chimeric hepatitis B virus/hepatitis C virus envelope proteins elicit broadly neutralizing antibodies and constitute a potential bivalent prophylactic vaccine. Hepatology.

[68] Correia BE, Bates JT, Loomis RJ (2014). Proof of principle for epitope-focused vaccine design. Nature.

[69] Yassine HM, Boyington JC, Mctamney PM (2015). Hemagglutinin-stem nanoparticles generate heterosubtypic influenza protection. Nat Med.

[70] Xu K, Acharya P, Kong R (2018). Epitope-based vaccine design yields fusion peptide-directed antibodies that neutralize diverse strains of HIV-1. Nat Med.

[71] He L, Cheng Y, Kong L (2015). Approaching rational epitope vaccine design for hepatitis C virus with meta-server and multivalent scaffolding. Sci Rep.

[72] Pierce BG, Boucher EN, Piepenbrink KH (2017). Structure-based design of hepatitis C virus vaccines that elicit neutralizing antibody responses to a conserved epitope. J Virol.

[73] Cowton VM, Owsianka AM, Fadda V (2021). Development of a structural epitope mimic: an idiotypic approach to HCV vaccine design. NPJ Vaccines.

[74] Sesterhenn F, Yang C, Bonet J (2020). De novo protein design enables the precise induction of RSV-neutralizing antibodies. Science.

[75] Brasher NA, Eltahla AA, Underwood A (2020). B cell immunodominance in primary hepatitis C virus infection. J Hepatol.

[76] Pestka JM, Zeisel MB, Blaser E (2007). Rapid induction of virus-neutralizing antibodies and viral clearance in a single-source outbreak of hepatitis C. Proc Natl Acad Sci U S A.

[77] Thimme R (2021). T cell immunity to hepatitis C virus: lessons for a prophylactic vaccine. J Hepatol.

